# Editorial: Rising stars in renal endocrinology: 2021

**DOI:** 10.3389/fendo.2023.1159850

**Published:** 2023-03-02

**Authors:** Juhong Yang, Baocheng Chang

**Affiliations:** National Health Council (NHC) Key Laboratory of Hormones and Development, Tianjin Key Laboratory of Metabolic Diseases, Chu Hsien-I Memorial Hospital & Tianjin Institute of Endocrinology, Tianjin Medical University, Tianjin, China

**Keywords:** type 2 diabetes, diabetic nephropathy, anemia, kidney fat, autonomous systems

There are 141 million adults with diabetes in China, accounting for 25% of the total number of diabetes patients in the world. Since 2011, diabetes related CKD has become the primary cause of CKD inpatients in China which bring serious effect on national health (1). In the tropic of the Rising Stars in Renal Endocrinology, we invited four professors to present their newly findings in DKD. Our aims was to introduce the new data of DKD burden in China as well as new findings of DKD risk factors from large clinical studies in the intention of arousing more attention and efforts to stop DKD.

Pan et al. analyzed the trends in diabetes-related CKD burden in China from 1990 to 2019, using the latest death and disability-adjusted life years (DALYs) data from the Global Burden of Disease study (GBD) 2019 study. They found that progress had been achieved in CKD in diabetes in China, as reflected by the decrease of age-standardized deaths and DALYs in 2019 as compared to 1990. However, because of the large population of diabetes, the deaths and DALYs were greatly increased in diabetes-related CKD compared with 30 years ago. In 2019, 76.03 (58.24-95.61) thousands of death and 2.13 (1.65–2.67) million DALYs were attributable to diabetes-related CKD.

In our previous study, we have set-up a DKD risk prediction model in a meta-analysis using 20 cohorts including 41,271 patients with type 2 diabetes (2). We have found that age, BMI, smoking, diabetic retinopathy, hemoglobin A1c, systolic blood pressure, HDL cholesterol, triglycerides, UACR, and eGFR are important risk factors of DKD. Pan et al. found that males had a greater burden in both deaths and DALYs since 1990 and this gender disparity was widened over the past three decades. This may be due to gender differences in health behaviors such as smoking and alcohol use. However, there are also other important risk factors of DKD including anemia (Xie et al.), renal fat deposition (Shen et al.) and autonomic neuropathy (Zeng et al.) as are presented in three other studies of our topic which all deserve great attention.

In patients with diabetes, anemia was usually considered as a consequence of DKD caused by inappropriate response to erythropoietin (EPO), chronic inflammation, and renal interstitial fibrosis (3). However, different results were found in the study by Xie et al. They included 2570 in-patients with type 2 diabetes with a median follow-up period of 2.75 years, and found that 22.44% of diabetic patients without DKD had anemia. This results were in line with a small study with a median of 7-year-follow-up: 10 out of 62 were found anemia in diabetic patients without kidney damages (4). Therefore anemia may not only be the consequence of DKD, but also a contributor to DKD. Moreover, Xie et al. found that anemia was an risk factor for rapid eGFR decline (exceed -5 ml/min per 1.73 m2 per year) independent of confounding factors in the logistic regression analysis.

In the study by Shen et al., fat deposited in renal parenchyma were measured in 189 diabetic patients using 3.0-T MRI. They found that after adjustment for potential confounders including anthropometric, metabolic factors, and other fat indicators, patients in the highest tertile of renal fat fraction had a significantly increased risk of CKD (defined as eGFR<60 mL/min/1.73m2) than those in the lowest tertile, and the area under the ROC curve was 0.836 (0.765–0.907).

Zeng et al. analyzed the relationship between heart rate variability and DKD in 747 T2DM patients. They divided all patients into four groups according to their risk of DKD progression based on the Kidney Disease: Improving Global Outcomes guidelines. They found that the reduction of heart rate variability was closely related to the increased risk of DKD progression. Compared with the low risk group, high-risk group had significantly lower heart rate variability. In accordance to this, a follow-up study reported that cardiac autonomic neuropathy predicted incident kidney disease in adolescents with type 1 diabetes (5).

Therefore, to decrease the great burden caused by DKD, based on the results of these four studies in our topic, early screening and intervention of anemia, renal fat deposition and cardiac autonomous neuropathy may be help in DKD control in addition to the control of traditional risk factors such as blood glucose, plasma lipids and blood pressure. However, further longitudinal and larger sample size studies are still needed to ensure the association between the these risk factors and CKD progress in diabetic patients.

## Author contributions

JY and BC contribute equally to the draft and writing of the paper. All authors contributed to the article and approved the submitted version.
